# Expression profiling of human milk derived exosomal microRNAs and their targets in HIV-1 infected mothers

**DOI:** 10.1038/s41598-020-69799-x

**Published:** 2020-07-31

**Authors:** Muhammad Atif Zahoor, Xiao-Dan Yao, Bethany M. Henrick, Chris P. Verschoor, Alash’le Abimiku, Sophia Osawe, Kenneth L. Rosenthal

**Affiliations:** 1 Department of Pathology & Molecular Medicine, McMaster University, MDCL 4019, 1280 Main Street West, Hamilton, ON L8S 4K1 Canada; 2 McMaster Immunology Research Center, McMaster University, Hamilton, ON Canada; 3 Evolve Biosystems, Davis, CA USA; 4 Department of Food Science and Technology, University of Nebraska, Lincoln, NE USA; 5 McMaster Institute for Research on Aging, McMaster University, Hamilton, ON Canada; 6 Institute of Human Virology-Nigeria, Plateau State, Abuja, 93000 Nigeria; 7 Institute of Human Virology, University of Maryland, Maryland, USA; 8 Present Address: Toronto Center for Liver Disease, Toronto General Hospital Research Institute (TGHRI), University Health Network, MaRS-Princess Margaret Cancer Research Tower 10-401, 101-College St., Toronto, ON M5G 1L7 Canada

**Keywords:** Infectious diseases, HIV infections

## Abstract

Despite the use of antiretroviral therapy (ART) in HIV-1 infected mothers approximately 5% of new HIV-1 infections still occur in breastfed infants annually, which warrants for the development of novel strategies to prevent new HIV-1 infections in infants. Human milk (HM) exosomes are highly enriched in microRNAs (miRNAs), which play an important role in neonatal immunity. Furthermore, HM exosomes from healthy donors are known to inhibit HIV-1 infection and transmission; however, the effect of HIV-1 on HM exosomal miRNA signatures remains unknown. In this study, we used nCounter NanoString technology and investigated miRNAs expression profiles in first week postpartum HM exosomes from HIV-1 infected and uninfected control mothers (n = 36). Our results indicated that HIV-1 perturbed the differential expression patterns of 19 miRNAs (13 upregulated and 6 downregulated) in HIV-1 infected women compared to healthy controls. DIANA-miR functional pathway analyses revealed that multiple biological pathways are involved including cell cycle, pathways in cancer, TGF-β signaling, FoxO signaling, fatty acid biosynthesis, p53 signaling and apoptosis. Moreover, the receiver operating characteristics (ROC) curve analyses of miR-630 and miR-378g yielded areas under the ROC curves of 0.82 (95% CI 0.67 to 0.82) and 0.83 (95% CI 0.67 to 0.83), respectively highlighting their potential to serve as biomarkers to identify HIV-1 infection in women. These data may contribute to the development of new therapeutic strategies in prevention of mother-to-child transmission (MTCT) of HIV-1.

## Introduction

Devastating statistics indicate more than 110,000 children die each year from HIV-1/AIDS related disorders and over 15,000 children are newly infected with HIV-1 every month^1^, which make up a substantial proportion of the approximately 40 million people are currently living with HIV-1 worldwide^1,2^. Indeed mother-to-child transmission (MTCT) of HIV-1 is an important source of HIV-1 infection in infants and can occur in utero, during delivery and through breastfeeding. Prophylactic strategies have greatly reduced the risk of MTCT HIV transmission from 35% to less than 5%; however, new HIV-1 infection in infants who are breastfed by HIV-1 positive mothers breastfeeding remains a major source of pediatric HIV-1 infection. Paradoxically, an intervention that promotes exclusive breastfeeding, regardless of HIV status of the mother shows decreased vertical HIV transmission rates compared to infants who are non-exclusively breastfed^1,3^. The mechanisms underlying this phenomenon remain unknown; however, data presented here may provide new insights into potential preventative and therapeutic strategies that may be useful for both infants and adults in the future^4^.

Importantly most of the infants born to HIV-1 positive women do not become infected themselves, which is potentially attributed to the anti-HIV-1 molecules present in human milk (HM)^5,6^. Previous work has shown specific HM exosomes, but not the plasma derived exosomes, had a protective effect by inhibiting HIV-1 infection of monocyte derived dendritic cells (MDDCs) and subsequent transfer to CD4+ T-cells. These data suggest that HM derived exosomes reduce the risk of MTCT of HIV-1 through breastfeeding^7^. Exosomes are nanosized intraluminal extracellular vesicles secreted by a variety of cells that are present in almost all biological fluids. Moreover, they are specialized in long distance intracellular communication and facilitating the transfer of nucleic acids such as messenger RNAs (mRNAs) and microRNAs (miRNAs) for subsequent expression in target cells in a highly regulated and efficient manner^8,9^. miRNAs are small, single-stranded, non-coding endogenous RNAs that suppress the target gene expression through translational repression^10–12^ and in this manner, play a key role in diverse biological processes including proliferation, differentiation and apoptosis^13^. Since miRNAs exist in plasma, urine, milk and other body fluids^14^, they hold great promise to serve as useful biomarkers in various diseases including cancer^15–24^.

Identification of HM exosomal miRNAs that play an important role in MTCT HIV-1 infection could be critical to our understanding of transmission; however, no studies exist that can document the expression profile of HM exosomes from HIV-1 infected women. Here, we investigated the expression profile of HIV-1 associated miRNAs in HM samples collected from HIV-1 infected women and identified a total of 19 miRNAs (fold change > 1.3; *P* < 0.05) which were differentially expressed in women infected with HIV-1 and targeted the cellular genes that are involved in multiple biological pathways including cell cycle, pathways in cancer, viral carcinogenesis, adherens junctions, TGF-β signaling, fatty acid biosynthesis, p53 signaling and apoptosis. Furthermore, our data indicated that two miRNAs, miR-630 and miR-378g could serve as biomarkers of HIV-1 infection. Taken together, these data identified important HM exosomal miRNAs which could be exploited in future studies for monitoring HIV-1 status in infected mothers as well as their potential role in the prevention of MTCT in infants.

## Materials and methods

### Human subjects

HIV-1-uninfected healthy control and HIV-1-infected women were recruited from the Plateau State, Nigeria to participate in the current study. All women were sampled during their voluntary ‘healthy’ research visits as per the cohort protocol^25^, and therefore, were not acutely ill at the time of sample collection. Women who were included in the study, were not taking medications other than ART or vitamin supplements intra- or post-partum and did not receive an epidural intra-partum. In addition, women were excluded if they had caesarean sections or they were diagnosed with mastitis post-partum.

### Sample acquisition and preparation

HM samples were self-collected into sterile tubes within the first week and at one, three, and six months post-partum, and immediately shipped on ice for processing in our laboratory. The samples were separated into lipid, skim milk supernatant, and cellular fractions and stored at − 80 °C and liquid nitrogen, respectively as previously described^26,27^.

### Exosome isolation from human milk

Exosomes were isolated from the skim milk supernatants using the Total Exosomes Isolation reagent (from other body fluids) as per manufacturer’s recommendations (Thermo Fisher, Canada). Briefly, 500 µl volume of each HM sample was centrifuged at 2000×*g* for 10 min (1st spin). Without disrupting the pellet, supernatant was transferred to a new tube and centrifuged again at 10,000×*g* for 30 min (2nd spin). The supernatant was transferred to a new tube and centrifuged at 10,000×*g* for 10 min (3rd spin). To the clear supernatant, 500 µl of 1 × PBS and 500 µl of exosome isolation reagent was added, vortex-mixed and incubated for 30 min at room temperature. After, incubation, the samples were centrifuged at 10,000×*g* for 10 min and the supernatant was removed carefully and discarded. The exosomes in the pellets were dissolved in 50 µl of exosome resuspension buffer (Thermo Fisher, Canada), vortex-mixed and again centrifuged at 10,000×*g* for 5 min at room temperature. Without disturbing the non-organic particulate matter in the pellet, the supernatants containing the purified HM exosomes were transferred to a new tube and stored at – 20 °C until further use.

### Transmission electron microscopy (TEM)

HM derived exosomes morphology was evaluated by TEM through negative staining as described^28^. Briefly, HM exosomes were placed onto formvar grids, fixed with 2.5% glutaraldehyde, and contrasted with 1% uranyl acetate and finally visualized with a JOEL-1200EX transmission electron microscope located at McMaster Electron Microscopy facility. The images with × 40,000–× 300,000 magnifications were taken using AMTV600 computer program.

### Western blotting

Exosomes were isolated from the HM samples as described above. Protein fraction was isolated, quantified using DC™ protein assay kit (Bio-Rad) and run on SDS-PAGE gel. Western blot analysis was performed with the primary antibody against CD81 (sc-166029; Santa Cruz) and HRP-labeled goat anti-mouse IgG 1706516 (Bio-Rad) as secondary antibody as described^26,27^.

### Exosome RNA isolation

Total RNA was extracted from the HM exosomes using Total Exosome RNA and Protein Isolation Kit as per manufacturer’s instructions (Invitrogen, Carlsbad, CA). Briefly, the isolated exosomes were dissolved in pre-warm 2 × denaturing solution followed by acid-phenol: chloroform extraction. The upper aqueous phase was precipitated with ethanol and total RNA was eluted with preheated (95 °C) elution buffer. The concentration of RNA was determined using the Nanodrop spectrophotometer (Nanodrop Technologies, Inc, Wilmington, Germany) as described^29^ and were stored at − 80 °C until further use.

### NanoString nCounter miRNA profiling and data analysis

Before processing of the NanoString chip, RNA samples were analyzed with the Agilent Bioanalyzer 2100 and the RNA 6000 Nano LabChip Kit (Agilent, CA, USA). RNA samples which did not pass the quality check were excluded and replaced with new RNA samples, thus, only high quality RNAs were processed for miRNA NanoString profiling. Exosomal miRNA expression profiling was performed using the nCounter Human ver 3.0 miRNA Panel on nCounter Analysis System (NanoString Technologies) as described^30^. A total of three cartridge chips were run at the same time each consisting of 12 samples (9 HIV-1 positive and 3 negative control per chip). For data analysis, HIV-1 positive and control samples were separately pooled. Raw NanoString counts were pre-processed and differential counts derived using the R package ‘edgeR’ (PMID: 19910308) as described^29,31^. Briefly, counts were normalized using trimmed mean of M-values (TMM) method and miRNA that were less than the geometric mean of negative control probes for more than half of the samples were removed; the final miRNA count for differential expression analysis was 267. Differential expression between groups was calculated using the function exactTest, which is analogous to Fisher’s exact test, but adapted for overdispersed data (PMID: 19910308). Adjusted p-values were derived using Benjamini-Hochberg’s procedure for controlling false discovery rate. In order to predict the role of these miRNAs as a biomarker, Receiver Operating Characteristic (ROC) curves were generated for the top five miRNAs and their areas under the curve (AUC) were calculated using the R package “plotROC” (*P* < 0.001).

### In-silico bioinformatic analysis

For functional classification of miRNAs, DIANA-mirPath v3.0 was used for Kyoto Encyclopedia of Genes and Genomes (KEGG) pathway and Gene Ontology (GO) annotation analyses as described^32^. DIANA-Tarbase and “Pathways Union” options were selected to perform a KEGG pathway analysis using this database of experimentally validated targets. For GO analysis, miRNAs belonging to specific GO categories based on the experimental findings with “Categories Union” was conducted. The significance of each functional annotation term was generated using a modified Fisher’s exact test with *P*-value threshold of < 0.05 for KEGG pathways and < 1e−20 for GO analysis, respectively as described^32^. The candidate target genes of the identified miRNAs were predicted using TarBase v8.0^33^. The untranslated region (UTR) location was predicted by TargetScan v7.2^34^. In order to identify the potential interactions of the differentially expressed miRNAs in HM from HIV-1 infected women, miRNA-mRNA Network analysis was performed using Network Analyst software^29^ where miRNA to gene interaction data were collected from well-annotated databases such as miRTarBase v7.0, TarBase v7.0 and miRecords as described^35^. For degree, betweenness and shortest Path, “all but miRNA nodes” filter option was selected for the analysis.

### Ethics approval and consent to participate

Written as well as informed consent for the collection of demographics, behavioral data, and biological samples were obtained from all study participants. The study was approved by the McMaster Research Ethics Board (REB Approval #08-176), CCI of Children’s Hospital, Los Angeles, the institutional review boards of the University of Manitoba Hospital ethical review committee, University of Maryland Baltimore and Plateau State Specialist Hospital Nigeria Institutional Review Boards as described previously^25,27^. All clinical investigations were conducted according to the principles of the Helsinki Declaration.

## Results

### Clinical characteristics of women participants

**Figure 1:**
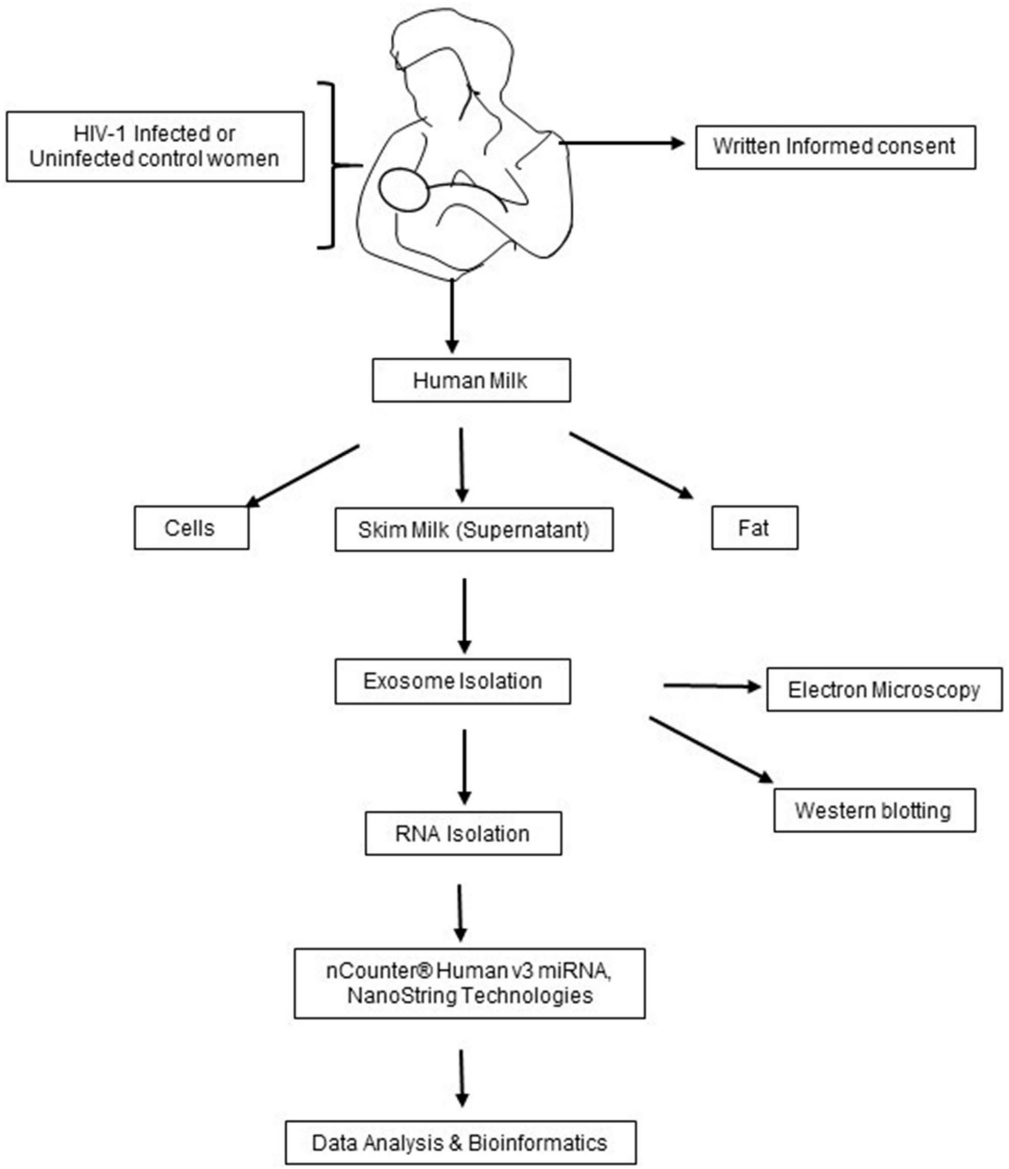
Schematic layout of the methodology adopted for Human Milk Exosomal miRNA Profiling. Human milk samples were collected from HIV-1 infected and uninfected control women from Nigeria. The samples were processed for exosome isolation and confirmed either by electron microscopy or by Western blotting using Exosomal marker protein CD81. Exosomal RNAs were extracted, subjected to integrity check by Bioanalyzer and run for miRNA profiling using nCounter Nanostring Human v3.0. The data were analyzed, and targets were predicted.

HIV-1 infected and uninfected women were recruited from the Plateau State, Nigeria as described previously^27,36^ A total of 36 HM samples from the first week postpartum (27 HIV-1 positive and 9 HIV-1 negative as controls) were processed for HM exosome miRNA profiling, as shown in the study layout in Fig. 1. The characteristics of the study population included in the current analyses are presented in Table 1. Of the 27 HIV-1 positive mothers, 22 were infected with HIV-1 for 4–15 years whereas, 5 women were infected with HIV-1 for only 3 years. All HIV-1 positive women were receiving ART according to the regimen set by Nigerian Government and the WHO^37^ and had CD4+ count ≤ 300/mm^3^ with undetectable viral load. It was impossible to obtain samples from ART-naïve HIV-1 positive women. Number of years on ART were counted, the day a woman was diagnosed positive for HIV-1 and placed on ART. As shown in Table 1, 13 out of 22 HIV-1 positive women showed high infant mortality compared to uninfected. Additionally, infants born to HIV-1 positive women were found to be stunted and underweight similar to what has been described previously^25^.

**Table 1:** Clinical characteristics of HIV-1-infected and uninfected mothers.

Sr. no.	ID no.	Status	Age	HIV status (duration till 2017)	ART (years)	No. of previous pregnancies	Live babies*
1	N-0379-M	Control	30	–	–	0	0
2	N-0380-M	Control	30	–	–	3	3
3	N-0382-M	Control	26	–	–	1	0 (1)
4	N-0353-M	Control	30	–	–	3	3
5	N-0356-M	Control	31	–	–	0	0
6	N-0364-M	Control	37	–	–	7	7
7	N-0369-M	Control	31	–	–	0	0
8	N-0371-M	Control	29	–	–	3	3
9	N-0374-M	Control	32	–	–	2	1 (1)
10	N-0230-M	HIV-1	30	2014 (3 years)	3	0	0
11	N-0231-M	HIV-1	35	2009 (8 years)	8	9	4 (5)
12	N-0232-M	HIV-1	44	2006 (11 years)	11	5	3 (2)
13	N-0236-M	HIV-1	35	2011 (6 years)	6	3	3
14	N-0247-M	HIV-1	27	2013 (4 years)	4	2	2
15	N-0248-M	HIV-1	30	2014 (3 years)	3	3	3
16	N-0249-M	HIV-1	28	2011 (6 years)	6	2	0 (2)
17	N-0251-M	HIV-1	29	2006 (11 years)	11	5	4 (1)
18	N-0254-M	HIV-1	29	2008 (9 years)	9	1	1
19	N-0130-M	HIV-1	31	2013 (4 years)	4	2	2
20	N-0138-M	HIV-1	29	2011 (6 years)	6	3	1 (2)
21	N-0140-M	HIV-1	39	2007 (10 years)	10	5	5
22	N-0160-M	HIV-1	36	2014 (3 years)	3	6	5 (1)
23	N-0164-M	HIV-1	24	2013 (4 years)	4	2	0 (2)
24	N-0178-M	HIV-1	31	2007 (10 years)	10	3	2 (1)
25	N-0181-M	HIV-1	28	2011 (6 years)	6	1	1
26	N-0192-M	HIV-1	34	2008 (9 years)	9	0	0
27	N-0196-M	HIV-1	29	2012 (5 years)	5	0	0
28	N-0207-M	HIV-1	31	2006 (11 years)	11	7	5 (2)
29	N-0211-M	HIV-1	35	2008 (9 years)	9	3	2 (1)
30	N-0222-M	HIV-1	29	2010 (7 years)	7	0	0
31	N-0226-M	HIV-1	30	2009 (8 years)	8	2	2
32	N-0233-M	HIV-1	34	2006 (11 years)	11	4	4
33	N-0234-M	HIV-1	26	2014 (3 years)	3	0	0
34	N-0238-M	HIV-1	30	2014 (3 years)	3	7	5 (2)
35	N-0239-M	HIV-1	32	2008 (8 years)	8	4	2 (2)
36	N-0240-M	HIV-1	28	2002 (15 years)	15	2	1 (1)

*Number of deceased are shown in parenthesis.

### Milk exosome characterization and RNA quality check

**Figure 2:**
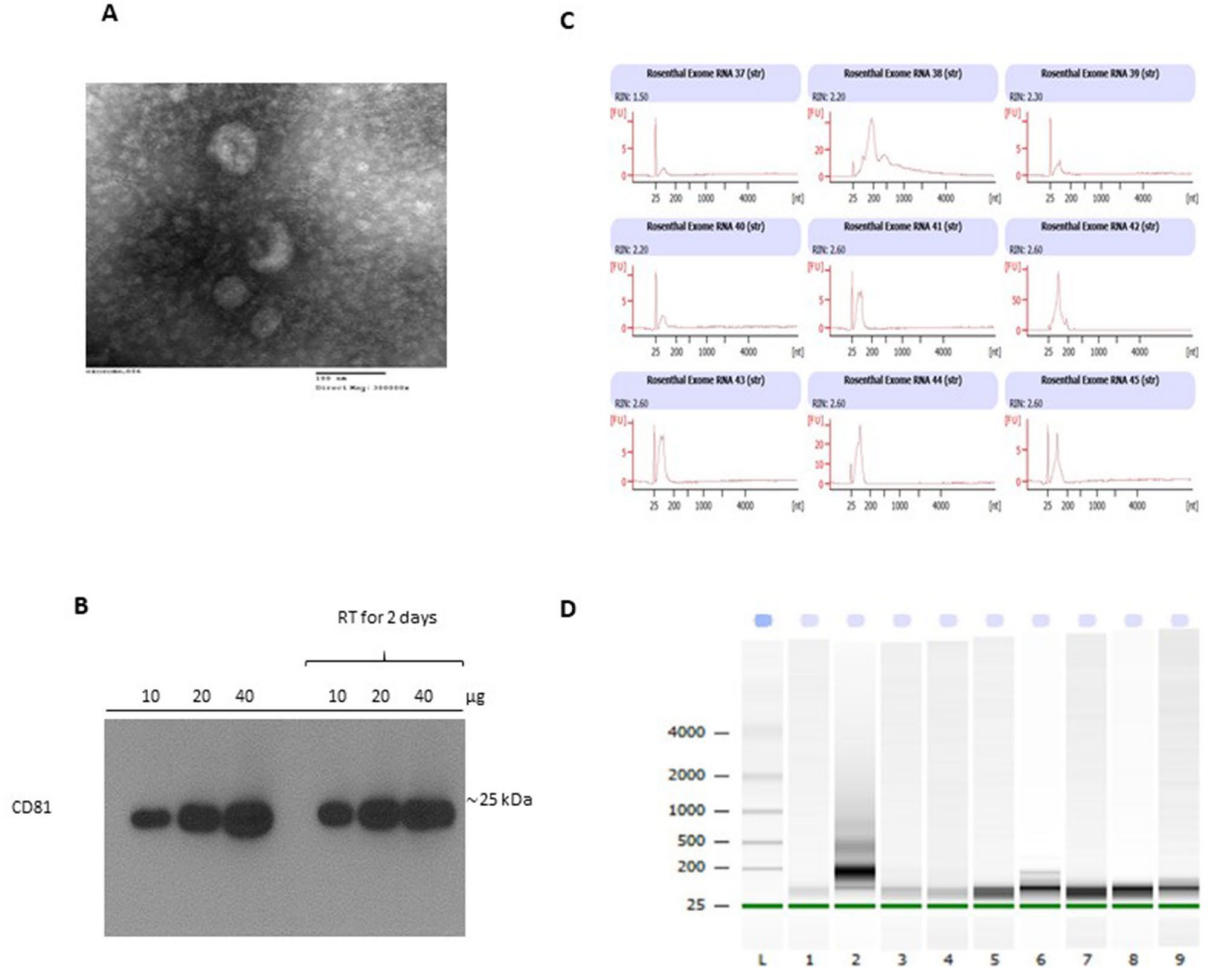
Human Milk Exosome characterization and RNA isolation. (**A**) Transmission electron micrograph of human milk exosomes demonstrates small vesicles with sizes ranging from 30 to 100 nm in diameter (Magnification: × 300,000; Scale bar 100 nm) (**B**) Western blotting for exosome-associated marker protein CD81. 10, 20, and 40 µg of proteins from either freshly isolated or human milk exosomes kept at room temperature (RT) for 2 days were loaded (**C**) Electropherogram and (**D**) gel images of representative RNAs extracted from human milk exosomes run on Agilent 2100 Bioanalyzer. Lanes 1–9 correspond to 37–45 samples; *L* Ladder.

Exosomes were isolated from individual HM samples and confirmed by TEM. HM derived exosomes were 30–100 nm in size and largely spherical in shape (Fig. 2A). HM derived exosomes either freshly isolated or kept at room temperature for two days were confirmed using protein marker CD81 in a western blot analysis (Fig. 2B, Suppl Fig. S1). Next, exosomal RNA was isolated from HIV-1 positive and negative HM samples with an average yield of 40 ng/µl. The isolated RNA showed distinctive spikes of noncoding RNA bands at > 25 nucleotides (Fig. 2C,D).

### Identification of HM derived exosomal miRNAs from HIV-1 infected women

**Figure 3:**
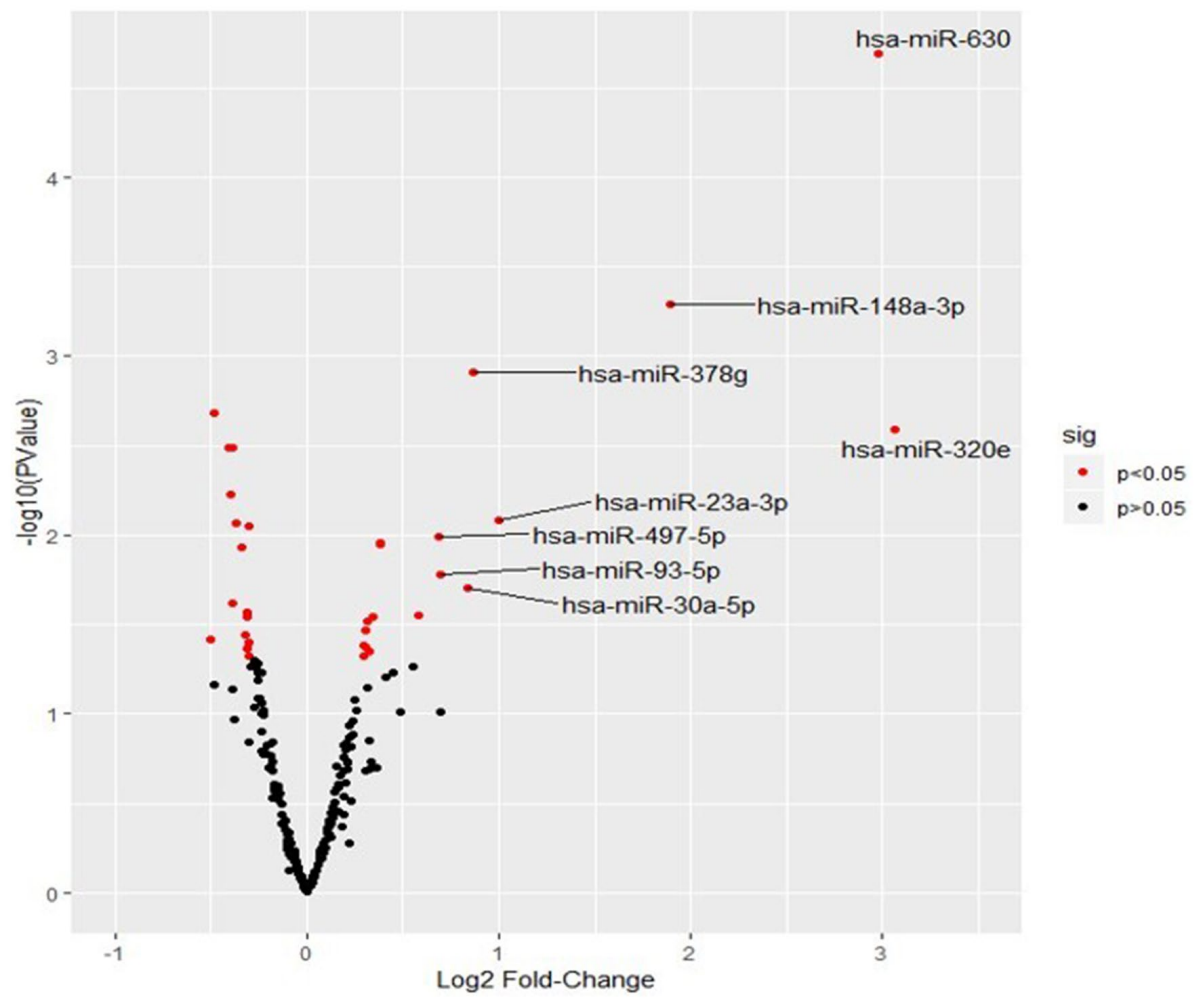
Differential expression of human milk exosome miRNAs in HIV-1 infected women. The differential expression profile of human milk exosomal miRNAs is shown as a volcano plot which demonstrates fold change versus significance (*P* < 0.05) to exhibit differences in the miRNA expression between HIV-1 infected and uninfected control women. The X-axis of the plot shows log-base two-fold change whereas Y-axis shows the log *P*-value. Red color indicates the level of significance (*P* < 0.05).

Expression of 41 miRNAs were significantly different in HIV-1 positive compared to negative women (Fig. 3; *P* < 0.05). A complete list of all 41 miRNAs is shown in Suppl Table S1. Specifically 13 miRNAs were significantly upregulated (*P* < 0.05; FC > 1.3; Table 2) including hsa-miR-320e; hsa-miR-630; hsa-miR-148a-3p; hsa-miR-23a-3p; hsa-miR-378g; hsa-miR-30a-5p; hsa-miR-93-5p; hsa-miR-497-5p; hsa-miR-200b-3p; hsa-miR-200a-3p; hsa-miR-16-5p; hsa-miR-1262 and hsa-miR-4516. Conversely 6 miRNAs were downregulated in HIV-infected breast milk compared to uninfected, including hsa-miR-422a; hsa-miR-644a; hsa-miR-520a-5p; hsa-miR-506-5p; hsa-miR-1257 and hsa-miR-1253 (Table 2). Upon further analyses, mothers living with HIV-1 for 3 and 4–15 years showed 28 (18 upregulated; 10 downregulated) and 17 (10 upregulated; 7 downregulated) differentially regulated miRNAs, respectively which were not significantly different in terms of fold change (Suppl Tables S2, S3). From hereon, the prefix hsa was removed from the miRNAs.

**Table 2:** Differentially expressed human milk exosomal miRNAs in HIV-1 infected mothers.

Sr. no	miRNA ID	Log fold change	Fold change	p-value	Adj. p-value	Regulation
1	hsa-miR-320e	3.06611	8.375124	2.57E−03	0.124605	Up
2	hsa-miR-630	2.979453	7.886873	2.03E−05	0.005408	Up
3	hsa-miR-148a-3p	1.896117	3.7221	5.14E−04	0.068612	Up
4	hsa-miR-23a-3p	1.003622	2.005028	8.26E−03	0.207538	Up
5	hsa-miR-378g	0.866517	1.823256	1.24E−03	0.110628	Up
6	hsa-miR-30a-5p	0.834559	1.783312	1.96E−02	0.308391	Up
7	hsa-miR-93-5p	0.693353	1.617037	1.68E−02	0.279933	Up
8	hsa-miR-497-5p	0.682571	1.604997	1.02E−02	0.207538	Up
9	hsa-miR-200b-3p	0.580053	1.494904	2.82E−02	0.350687	Up
10	hsa-miR-16-5p	0.555462	1.469639	5.44E−02	0.386215	Up
11	hsa-miR-422a	− 0.50323	1.41738	3.84E−02	0.381103	Down
12	hsa-miR-644a	− 0.47998	1.394723	2.08E−03	0.124605	Down
13	hsa-miR-200a-3p	0.445082	1.361391	5.88E−02	0.386215	Up
14	hsa-miR-520a-5p	− 0.40659	1.325552	3.27E−03	0.124605	Down
15	hsa-miR-506-5p	− 0.39559	1.315479	5.92E−03	0.197641	Down
16	hsa-miR-1262	0.386293	1.307031	1.13E−02	0.207538	Up
17	hsa-miR-4516	0.386214	1.306959	1.10E−02	0.207538	Up
18	hsa-miR-1257	− 0.38522	1.306057	3.23E−03	0.124605	Down
19	hsa-miR-1253	− 0.38439	1.305306	2.43E−02	0.350687	Down

### Top ten miRNAs and their potential target genes

Since miRNAs act by directly silencing and/or reducing the expression of target genes, we next predicted the validated target genes of the top ten differentially expressed miRNAs using a well characterized database (Tarbase v8.0)^33^. Our results indicated a total of 19895 interactions (Table 3). miR-16-5p was found to target the largest number of genes including IRF9, TLR4, TLR6 and JUN. miR-630 was shown to target BCL2, BCL2L11 and YAP1. miR-378g was shown to target 123 genes including SMAD2, CREBBP and WDR5. Interestingly, multiple miRNAs including miR-320e and miR-148a-3p targeted BCL2L11 and miR-630 and miR-497-5p both targeted BCL2L2. miRNAs, miR-30a-5p and miR-200b-3p, targeted NOTCH1, while miR-378g, miR-497-5p and miR-16-5p targeted SMAD2 (Table 3).

**Table 3:** Tarbase v8.0 based target genes of the top ten differentially expressed human milk exosomal miRNAs in HIV-1 infected mothers.

Sr. no.	miRNA ID	No. of interactions	Target genes (gene symbols)*	References
1	hsa-miR-320e	327	CDK6, MEGEA5, IGF2BP3, BTG2, WASF2, MINK1, SFPQ, PTBP1, BLMH, JUN, NUCKS1, CDK16, DYRK2, RTN4, CAND1, CLN6, MARCK5, RPL32, OAZ1, CSNK1A1, BAMBI, IRF3, DCAF7, ITGAV, BCL2L11, HNRNPU, DHX33, RNF10, UBE3C, WDR6, HERC1, NSD3	^38,39^
2	hsa-miR-630	3	BCL2, BCL2L2, YAP1	^40^
3	hsa-miR-148a-3p	1,481	DNMT1, IGF1R, IRS1, ITGB8, BCL2L11, DCAF7, RAB1B, USP28, CDKN1B, ZNF460, TNRC6A, JAR1D2, CDK1, ATP5E, GAND1, PRNP, PBXIP1, CAND1, PBXIP1, RAB1B, CCN1, NR1D2, DCAF7, USP28	^41–45^
4	hsa-miR-23a-3p	1744	ZNF91, CXCL12, LAMP1, IFNG, CDK17, CDK1, HDAC7, TCF20, MTMR2, TOP2B, VACN, TRIB1, UBL3, FUT9, TSNAX, TJP2, AHNAK, TNRC6A, ZNF107, CBX5, CREBZF, SESN3, UFM1, EIF2A, STX12	^13,45,46^
5	hsa-miR-378g	123	TAOK1, PPP1R37, VE2F1, SP4, CBLC1, MBP, PSMD8, PABPC3, BLOC1S6, WDR5, SMAD2, FKBP4, PIAS1, KCTD20, PSMD8, TNRC6C, PAPOLA, CREBBP, CDH1, TARBP2, HIVEP3	^42,47^
6	hsa-miR-30a-5p	2,808	TNRC6A, DDIT4, RHOB, E2F7, BRWD1, NDEL1, DHX36, FBXO45, RPA2, XRN1, SOCS1, PLA2G12A, IFNGR2, DCAF12, ATG12, ERLIN1, NOTCH1, BECN1P1, DDAH1, MAST4, AGO2, SOX4, IPO4, SLC20A1, RNF139, VAMP3, DDIT4, RHOB, ANKRA2, LCOR, ZBTB18, NDEL1, XPO1, XRN1, PFN2, SRSF7	^41,42,45,48^
7	hsa-miR-93-5p	2,227	CDKN1A, CSKN1A1, CLIP1, ANKIB1, BACH1, CLTC, ULK1, DZAPAP2, CCND1, PELI1, MOB1A, E2F1, PIK3R1, RAB22A, IRF9, TNKS2, MAPK1, TXN, TMEM138, USP31, RRM2, MKNK2, PFN2, PURA, ADAM9, ZNFX1, RUFY2, TXNIP, SEMA7A, JAK1, CNOT4, WEE1, BNIP2, IPO7, RAB5B	^42,43,47,49,50^
8	hsa-miR-497-5p	1,590	CCND1, CCNE1, E2F3, CDK6, ACTB, CCND3, CDk4, CDC25A, BTRC, TXNIP, FBXW7, ABI2, BTG2, WEE1, HSPA1B, ARL2, CSDE1, CCND1, CDCA4, BTG2, ATG9A, PDCD4, SESN3, SMAD2, NOTCH2, IGF1R, DDX6, MAPK8, PURA, TLK1, TACC1, HIPK2, BCL2L2	^45,47,51^
9	hsa-miR-200b-3p	1905	TCF7L1, ERBB21P, VAC14, RASSF2, NOTCH1, CDKN1B, AKAP11, CAB39, ANKRD42, ETS1, KRAS, YES1, TBP, XIAP, ZEB1, BCL2, NDFIP2, WEE1, JUN, RND3, ETS1, GLS, KDR, TOB1, NRBP1, FLT1, SMURF2, IRF9	^47,52–54^
10	hsa-miR-16-5p	7,687	TLR4, HIST1HIC, WEE1, DNAJB4, JUN, NUFIP2, CCND1, RRHGDIA, TNRC6A, LATS1, KIF21A, CDCA4, TLR6, DDX17, PSAT1, PRAGA, ODC1, SP1, CDK6, SMAD2, LAMP2, RBF217, XPO7, RANBP6, KPNA1, RNF217, XPO7, RANBP6, KPNA1, IRF9	^12,43,45,47^

*Some of the target genes out of total are shown here in the table.

### GO and KEGG pathways

**Figure 4:**
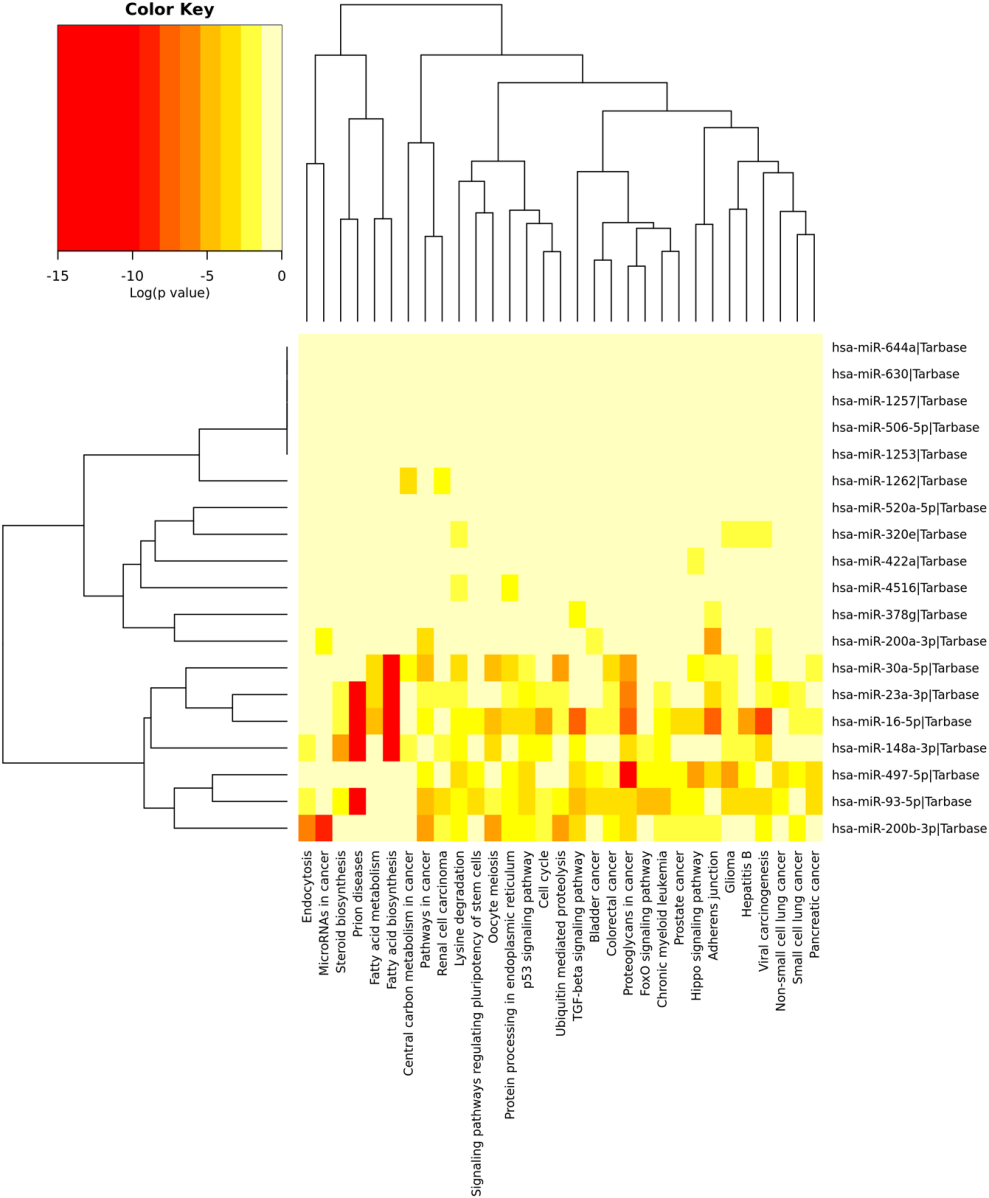
KEGG Pathway analysis of differentially expressed human milk exosome miRNAs. Differentially expressed exosomal miRNAs in HIV-1 infected human milk regulate multiple cellular pathways. Depicted here in the heatmap, significant pathways generated by DIANA-miRPath v3.0 software using Tarbase database are shown on the X-axis whereas miRNAs are shown on the Y-axis. The color code represents the log (*P*-value), with the most significant predicted miRNA-pathway interactions in red.

**Figure 5:**
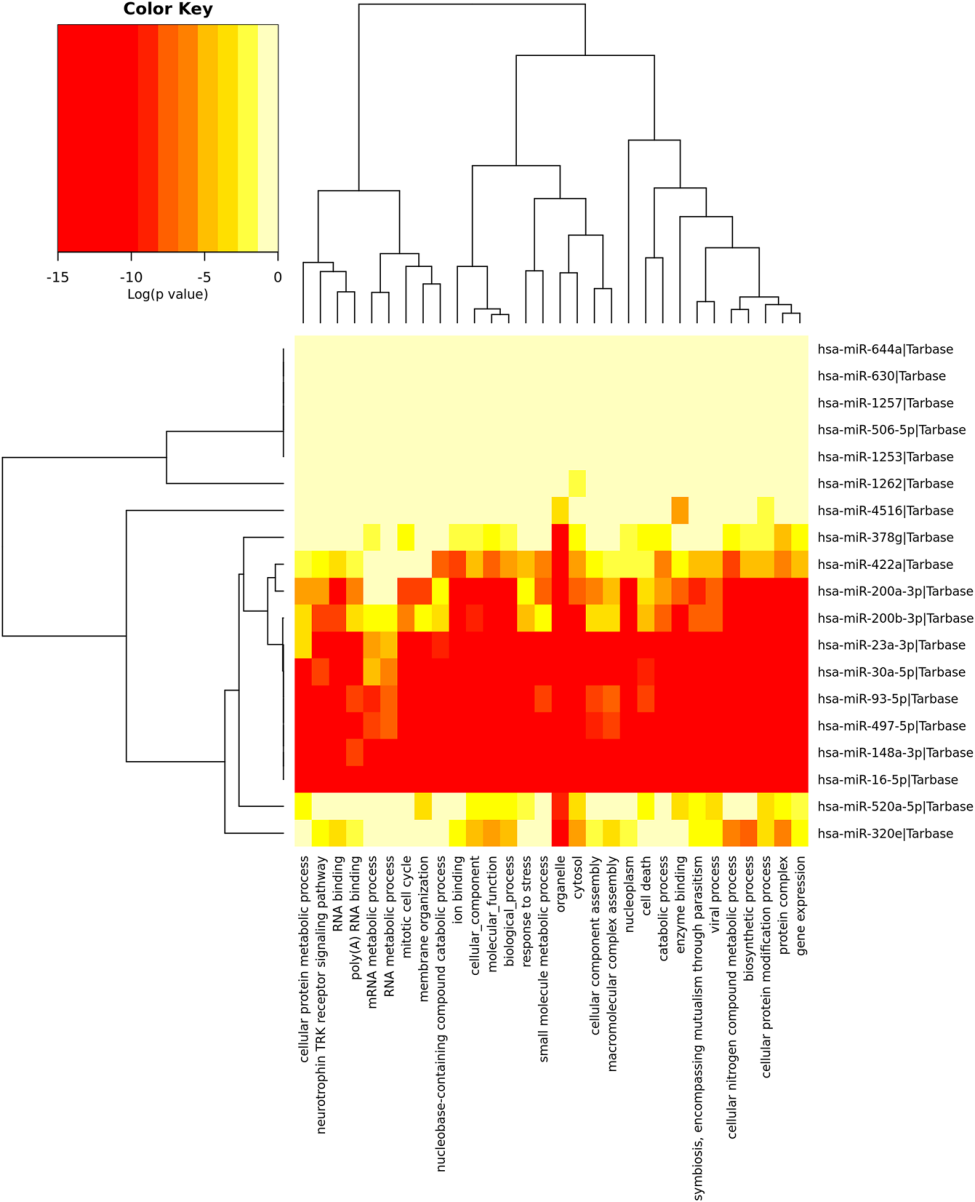
Gene Ontology Analysis of differentially expressed human milk exosome miRNAs. Gene Ontology of differentially expressed exosomal miRNAs in HIV-1 infected human milk is shown as heatmap which was created by DIANA-miRPath v3.0 software using Tarbase database. The heatmap enables similar miRNAs to cluster together in the same GO term. The color code represents the log (*P*-value), with the most significant GO term in red.

KEGG pathway analysis on the predicted targets led to the identification of 31 significant pathways in which the predicted miRNA targets were enriched (Fig. 4). Specifically, miRNA targets associated with HIV-1 belonged to multiple pathways such as pathways in cancer, viral carcinogenesis, adherens junctions, TGF-β, fatty acid biosynthesis, p53 signaling, cell cycle, pathways regulating pluripotency of stem cells and proteoglycans in cancer (Fig. 4). Next, we performed GO analysis to identify the biological processes associated with the miRNAs. A total of 30 GO biological processes were observed (Fig. 5). The highest enrichment GO terms targeted by these miRNAs included biosynthetic process followed by viral process, catabolic process, cell death, ion binding, membrane organization, mitotic cell cycle, RNA metabolic process, poly (A) RNA binding and neurotropin TRK receptor signaling pathway (Fig. 5).

### miRNA–gene interaction network

**Figure 6:**
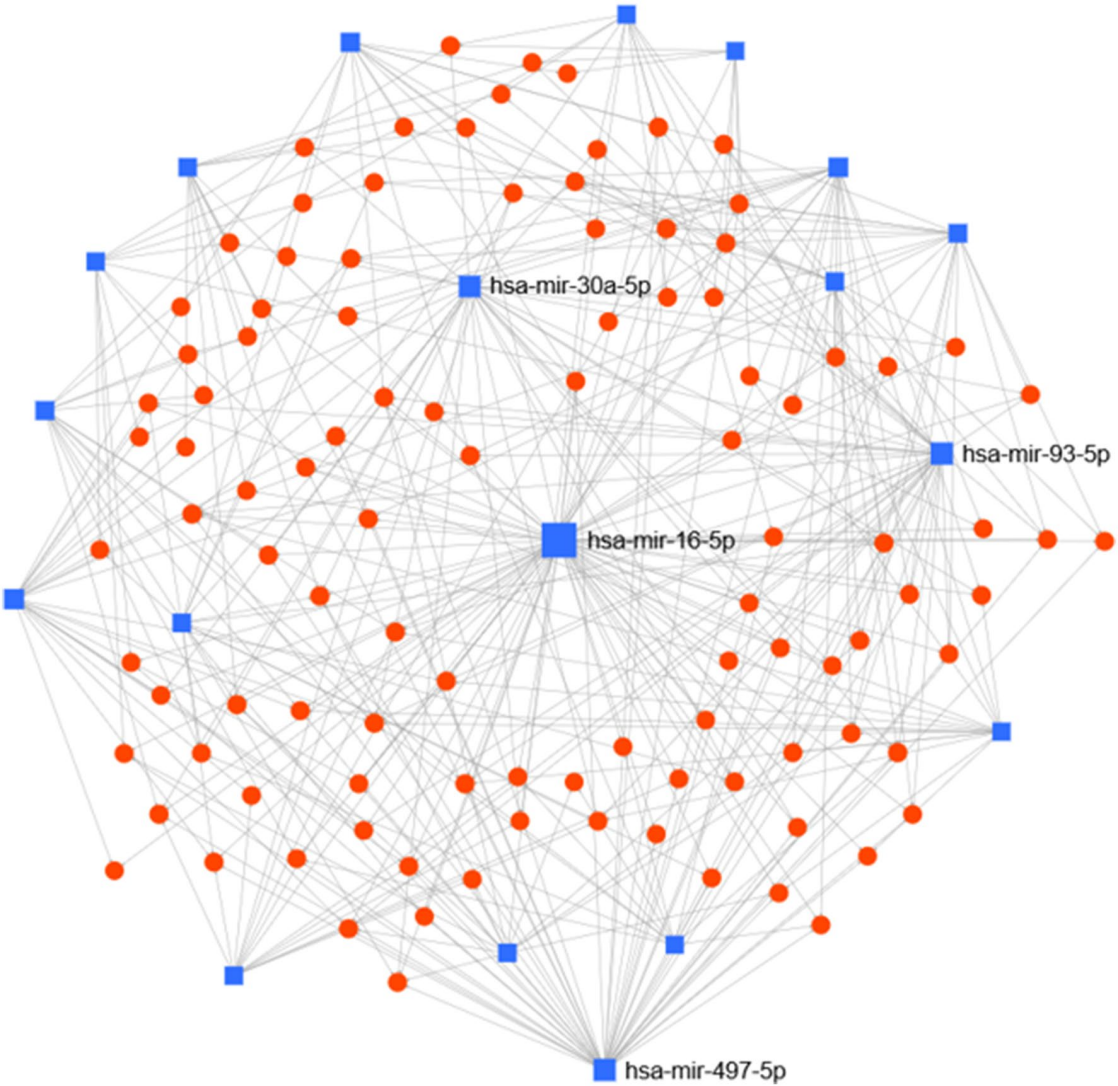
Network analysis of differentially expressed miRNAs and their target genes in HIV-1 infected human milk. Network display showing differentially expressed miRNAs (Fold change 1.3; *P* < 0.05) and their target genes in HIV-1 infected human milk. Cluster hubs shown in blue squares indicate miRNAs whereas red circles depict their target genes.

To understand the association of differentially expressed miRNAs in HIV-1 infected HM and their target proteins, miRNA-gene interaction network was generated using miRNet tool. The 19 differentially expressed miRNAs were uploaded into miRNet platform and miRNA-gene interactions were observed which generated 4190 target nodes and 6042 edges. Shortest path filter with “all but miRNA nodes” generated 124 nodes with 105 targets and 393 edges (Fig. 6). The top cluster hubs included miR-16-5p followed by miR-497, miR-93-5p, miR-30a-5p and miR-23a-5p. The biological functions were determined within the “reactome database” using the “hypergeometric test” algorithm and P-value < 0.05. Results showed that pre-notch transcription and translation (TP53, E2F3, AGO2, CCND1) was the top group followed by mitotic G1/S phase (Wee1, CDK6, CDKN1A, E2F3, PSMD11, CCND1, CCNE2), cyclin D associated events in G1 (E2F3, CDK6, CDKN1A, CCND1), EGFR signaling (EGFR, GRB2, HSP90AA1), Intrinsic pathway of apoptosis (BCL2, XIAP, TP53, DYNLL2) and cell cycle (BIRC5, CCND1, CDK6, CDKN1A, CCNE2, WEE1, TP53, PSMD11, PAFAH1B1, HSP90AA1, E2F3, TAOK1, FOXM1, CSNK2A1). Collectively, these results demonstrate the target genes of miRNAs perturbed by HIV-1 belong to multiple biological pathways.

### miR-630 and miR-378g as biomarkers of HIV-1 infection

**Figure 7:**
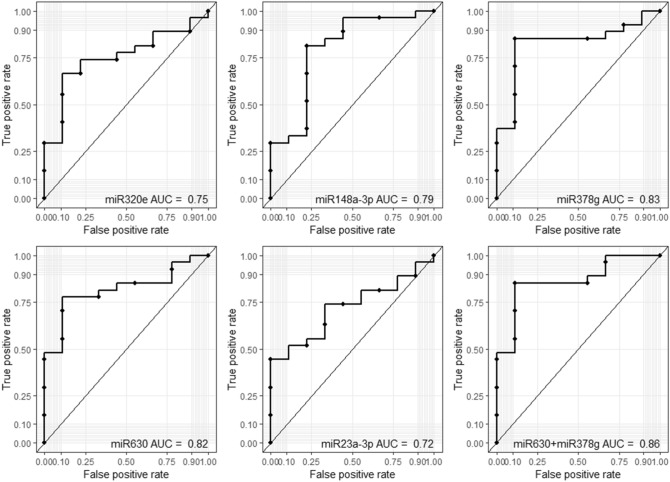
ROC curve analysis of human milk exosome miRNAs. ROC curves for the individual top five (miRNA-320e, miRNA-148a-3p; miRNA-378g, miRNA-630 and miRNA-23a-3p) or combined miRNAs (miRNA-630 and miRNA-378g) for discriminating HIV-1 infected women from healthy controls. *ROC* receiver operating characteristic, *AUC* area under the ROC curve.

In order to evaluate the utility of exosomal miRNAs in HM as a potential diagnostics, we performed ROC curve analysis of the top 5 miRNAs in discriminating HIV-1 infected women from healthy controls. This analysis showed that miR-320e, miR148a-3p, miR-378g, miR-630 and miR-23a-3p were substantially increased in HIV-1 infected breast milk and showed ROC AUC values of 0.75 (95% CI 0.58 to 0.75), 0.79 (95% CI 0.6 to 0.75), 0.83 (95% CI 0.67 to 0.83), 0.82 (95% CI 0.67 to 0.82) and 0.72 (95% CI 0.55 to 0.72) respectively (Fig. 7). Furthermore, when miR-630 and miR-378g were combined, it yielded ROC AUC of 0.86 (95% CI 0.72 to 0.86) (Fig. 7) suggesting that miR-630 and miR-378g could serve as biomarkers to distinguish HIV-1 infected HM from non-HIV-1 infected HM.

### Schematic model showing miR-378g mediated HIV-1 transactivator (TAR) binding protein 2 (TARBP2) depletion and its predicted role in HIV-1 infection

**Figure 8:**
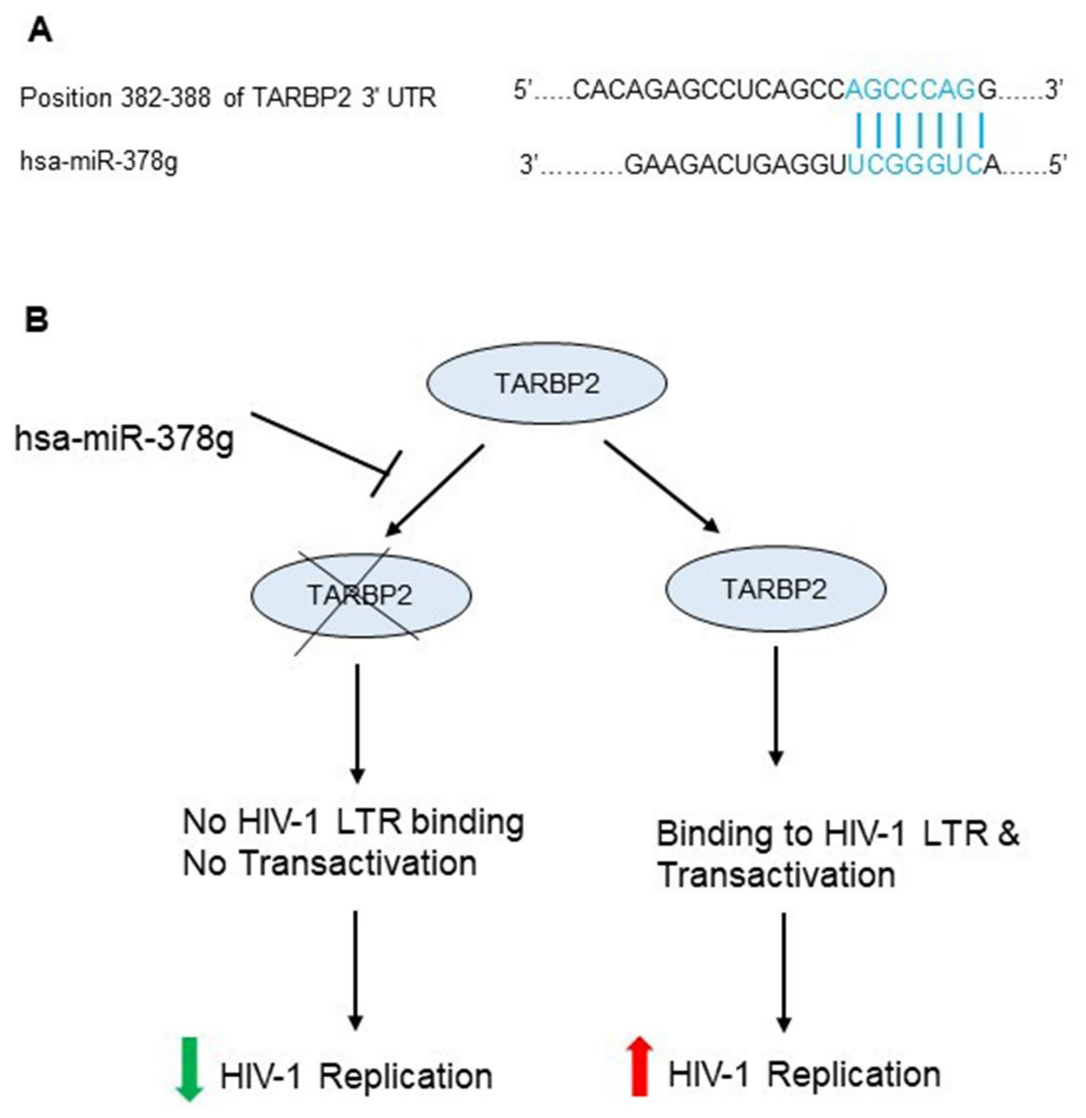
Schematic model showing miR-378g mediated TARBP2 depletion and inhibition of HIV-1 replication. (**A**) Target nucleotide sequence of 3′ UTR of Human TARBP2 (ENST00000552857.1) recognized by miR-378g as confidently annotated by TargetScan v7.2. Predicted consequential pairing of target region is shown in the top and miRNA-378g sequence in the bottom. (**B**) TARBP2, a cellular protein originally identified as a binding partner of HIV-1 LTR (transactivation response element found at both 5′ and 3′) and is well known to enhance HIV-1 expression and virus production, once depleted by host cellular miR-378g would presumably lower the HIV-1 expression and virus production^55^.

TargetScan predicts the targets of miRNAs by searching for the presence of conserved 8-mer, 7-mer and 6-mer sites that match the seed regions of each mRNA^13^. Using the search term HIV, we identified that miR-378g has one target site in 3′ UTR of TARBP2 (ENST 00000552857.1) from 382 to 388 (Fig. 8A). TARBP2 is known to promote HIV-1 LTR expression and viral production whereas its siRNA-mediated knockdown inhibits HIV-1 LTR expression and viral production^55^. A schematic of the hypothetical layout is shown in Fig. 8B, where we speculate that miR-378g mediated RNA interference would lower HIV-1 expression and viral production essentially as previously described^56^.

## Discussion

HIV-1 is known to cause dramatic changes in cellular miRNA expression profiles^57–60^, however its effect on HM derived exosomal miRNAs remains unknown. Here, we characterized miRNA expression profiles of HM exosomes derived from HIV-1 infected HM and showed that HIV-1 infection significantly altered the expression levels of exosomal miRNAs. Analysis of differentially expressed miRNAs by a gene ontology and KEGG pathway-based approach revealed several biological processes are affected by HIV-1 infection. Furthermore, we identified two dysregulated miRNAs that can potentially discriminate HIV-1 positive HM from uninfected HM, with good predictive power. Collectively, these data provide, for the first time, comprehensive insight into HM exosomal miRNA profiles involved during HIV-1 infection.

Our data are timely given exosomes have recently emerged as new players in HIV-1 infection, albeit their exact role in HIV-1 pathogenesis and transmission is not completely understood^20,59^. Importantly, exosomes may act at different levels of HIV-1 pathogenesis by modulating immune responses, infectivity or possibly activating latent viral reservoirs^9^. Indeed, the impact of exosomes on HIV-1 has been suggested as a potential strategy to cure HIV-1 infection and/or therapeutic^61^. Currently, only a few reports exist that demonstrate the immune-modulatory functions of HM derived exosomes^62^ which may partly be due to the methodological limitations in their isolation and purification^8^. In this study, we have provided a successful HM exosome isolation method that will potentially aid future studies related to HM derived exosome characterization and mechanism.

To gain more insight into HM derived exosomal miRNAs modulation by HIV-1, we performed NanoString miRNA profiling and showed that HIV-1 perturbed the expression levels of 19 miRNAs (FC > 1.3; *P* < 0.05; Table 2). Further, we identified 31 KEGG pathways potentially regulated by miRNAs including pathways in cancer, TGF-β, fatty acid biosynthesis, FoxO signaling, p53 signaling, cell cycle, pathways regulating pluripotency of stem cells and proteoglycans in cancer. miRNA-mRNA network showed differentially expressed miRNAs are linked to each other via their target genes. Furthermore, miRNA-mRNA network analysis, in addition to cell cycle, apoptosis identified the involvement of NOTCH and EGFR pathways.

A combination of two miRNAs (miR-630 and miR-378g) had 86% accuracy rate in predicting HIV-1 infection which may serve as a biomarker for segregating HIV-1 positive HM from uninfected HM. The stability of HM exosomes at room temperature also raises the possibility of their utility in initial screening processes prior to HIV-1 specific blood testing in low-to-middle income countries. Mothers living with HIV-1 for 3 and 4–15 years showed no significant fold change differences in miRNA expression levels (Suppl Tables S2, S3). Furthermore, the majority of women who participated in the study were carrying HIV-1 for greater than 5 years (Table 1), thus it is suggested these two miRNAs could be used to monitor AIDS progression in HIV-1 infected women. Indeed, our finding is in agreement with a previous study in which miR-630 was reported as a biomarker in chronic progressors of HIV-1^63^. Interestingly, our HM exosomal miRNA data from the South Africa cohort where HIV-1 infected women were carrying HIV-1 load < 1 year (data not shown) showed downregulation of these miRNAs identified in the current study and correlated with a recent report where it was shown that acute HIV-1 leads to downregulation of miRNAs^57^. Nonetheless, miRNA data shown here could be used for future studies to gain more insight into HIV-1 pathogenesis.

miRNAs are thought to be involved in mediating immune suppression, establishment of viral latency^59,64^ or suppressing HIV-1 replication via decreasing HIV-1 dependency factors^65^. Some of the miRNAs we have identified have previously been implicated in HIV-1 infection including miR-630, miR-4516, miR-16-5p, miR-378, miR-93, miR-23, miR-30a^57,63,66–69^ thus strongly suggesting the reliability of the NanoString data obtained in the current study. Further, it has been described that NanoString can perform miRNA profiling with digital precision and the results do not require further validation by another method^70^. miRNA-630 causes apoptosis by targeting BCL2, BCL2L2 and IGF-1R^71^ or maintains the apoptotic balance by targeting multiple modulators^72^. miR-15a/b, miR-16, miR-20a, miR-93, miR-106b have been shown to bind Pur-α and repress its expression^68^. Pur-α is a cellular partner for Tat regulatory protein of HIV-1 and facilitates its transcriptional activity^73,74^ and is required for HIV-1 infection in macrophages^68,75^. We found that Pur- α is a target of HM exosomal miR-93-5p and miR-497-5p. Whether HM exosomal miR-93-5p and miR-497-5p lower the R5-tropic HIV-1 infection of macrophages is not known but will be intriguing to investigate in future studies.

HM derived immunomodulatory factors are transferred from mothers to infants via breastmilk which include immunoglobulins, cytokines, chemokines, growth factors, hormones, lactoferrins and Toll-like receptors^26,27,76–80^. TGF-β is a major cytokine in HM that favors preferential MTCT of R5-tropic HIV-1^81,82^. miR-378g, miR-16-5p and miR-497-5p were found to target SMAD2 which has previously been shown to mediate TGF-β and regulate multiple pathways such as cell proliferation, apoptosis and cellular differentiation^81,83^. HIV-1 positive human skim milk fraction after heat inactivation and proteolytic digestion retains HIV-1 inhibitory activity and was shown to significantly inhibit oral HIV-1 transmission in-vivo^3^. Since, HM exosomal miRNAs are known to reach the systemic circulation^62^, these data suggest the HM derived exosome containing miRNAs reported herein may reach the fetal systemic circulation via breastmilk and play an important role in lowering MTCT of HIV-1^7^. Further future studies are required to elucidate the functional role of these HM exosomal miRNAs.

Using a consensus scoring approach, it has been shown that miR-378 targets HIV-1 envelope gene^67^. mir-378 family consist of 11 mature miRNA members according to miRbase database (www.mirbase.org) comprising of miR-378a-5p, miR-378a-3p, miR-378b, miR-378c, miR-378d, miR-378e, miR-378f, miR-378g, miR-378h, miR-378i and miR-378j; however, this study is the first to indicate the involvement of miR-378g in HIV-1 infection. Furthermore, prediction analysis suggested miRNA-378g targets a site located in 3′ UTR of TARBP2 from 382 to 388 nucleotides (Fig. 8), which is required for HIV-1 expression and virion production^55,84^. Interestingly, astrocytes are shown to be resistant to HIV-1 infection due to low endogenous levels of TARBP2^85^. TARBP2 was originally identified as a protein that binds to the 59 nucleotides conserved TAR element found at the 5′ and 3′ ends of all HIV-1 transcripts and enhances its translation and replication^56,84,86^. In addition, miR-378g was also found to target 4 confidently annotated sites in 3′ UTR of human HIV-1 enhancer binding protein 3 (HIVEP3) (ENST00000372583.1) located at 582–588, 2026–2032, 2208–2214 and 2580–2586 nucleotides (data not shown). Although, the role of HIVEP3 in HIV-1 replication is not clear, targeting HIVEP2 by miRNAs is known to reduce HIV-1 replication^87^. The effect of miR-378g on HIV-1 replication and MTCT must be investigated in future studies.

HIV-1 associated neurologic disease (HAND) occurs in more than 25% of HIV-1 infected patients who develop AIDS^88^. Previously, miR-4516 has been shown to be a biomarker of HAND in HIV-1 infected patients^66^. Our data showed that miR-4516 is upregulated in 4–15 years HIV-1 infected HM which may suggest HIV-1 infected women were HAND-asymptomatic or already had developed HAND. Although, our clinical data did not collect any neurologic symptoms in these infected women, it will be interesting to monitor the immune status as well as the temporal expression pattern of miR-4516 in future studies.

In conclusion, these data are the first to characterize the expression of HM exosomal miRNAs in HIV-1 infected HM. Given the use of current ART in HIV-1 positive mothers does not completely mitigate MTCT of HIV-1, interventions including the use of exosomal miRNA in addition to available ART may be required to prevent new infections of HIV-1 in infants. In this context, our data detailing HM exosome miRNAs could potentially be exploited to lower MTCT HIV-1 transmission. Moreover, the miRNAs reported herein may serve as potential biomarkers of HIV-1 infected HM.

## Supplementary information

Supplementary Information

Supplementary Figure S1

## Data Availability

The datasets reported and analyzed in the current study are available in NCBI Gene Expression Omnibus (GEO) repository and are accessible through GEO series accession number GSE143039.
