# Supplementary material for: Expression profiling of human milk derived exosomal microRNAs and their targets in HIV-1 infected mothers
Source: Sci Rep. 2020 Jul 31;10:12931. doi: 10.1038/s41598-020-69799-x (PMC7395778; doi:10.1038/s41598-020-69799-x)
Supplement: Supplementary file 1 — Supplementary Information [file 41598_2020_69799_MOESM1_ESM.docx]

**Supplementary Information**

**Expression Profiling of human milk derived exosomal MicroRNAs and their targets in HIV-1 infected mothers”**

Muhammad Atif Zahoor^1,2*†^ Xiao-Dan Yao^1,2^, Bethany M. Henrick^3,4^, Chris P. Verschoor^1,2,5^, Alash’le Abimiku^6,7^, Sophia Osawe^6^ and Kenneth L. Rosenthal^1,2^**^*^**

^1^Department of Pathology & Molecular Medicine, ^2^McMaster Immunology Research Center, McMaster University, Hamilton, ON, Canada; ^3^Evolve Biosystems, Davis, California, USA, ^4^Department of Food Science and Technology, University of Nebraska, Lincoln, Nebraska, USA, ^5^McMaster Institute for Research on Aging, McMaster University, Hamilton, ON, Canada; ^6^Institue of Human Virology-Nigeria, Plateau State, 93000 Nigeria, ^7^Institute of Human Virology, University of Maryland, Maryland, USA,

^*^Correspondence:

Muhammad Atif Zahoor, PhD, Department of Pathology & Molecular Medicine, McMaster University, MDCL 4019, 1280 Main Street West, Hamilton, Ontario, Canada L8S 4K1. Telephone (905) 525-9140 x22375; Email: [zahoorma@mcmaster.ca](mailto:zahoorma@mcmaster.ca)

Kenneth L. Rosenthal, PhD, Department of Pathology & Molecular Medicine, McMaster University, MDCL 4019, 1280 Main Street West, Hamilton, Ontario, Canada L8S 4K1. Telephone (905) 525-9140 x22375; Email: [rosenthl@mcmaster.ca](mailto:rosenthl@mcmaster.ca)

^†^Current Address: Toronto Center for Liver Disease, Toronto General Hospital Research Institute (TGHRI), MaRS-Princess Margaret Cancer Research Tower 10-401, University Health Network, 101-College St. Toronto, ON M5G 1L7 [atif.zahoor@uhnresearch.ca](mailto:atif.zahoor@uhnresearch.ca)

**Suppl Table 1: Differentially expressed human milk exosomal miRNAs in HIV-1 infected mothers**

| **Sr. No.** | **miRNA ID** | **Log Fold Change** | **Fold Change** | **p-value** | **Adj. p-value** | **Regulation** |
| --- | --- | --- | --- | --- | --- | --- |
| 1 | hsa-miR-320e | 3.06611 | 8.375124 | 2.57E-03 | 0.124605 | UP |
| 2 | hsa-miR-630 | 2.979453 | 7.886873 | 2.03E-05 | 0.005408 | UP |
| 3 | hsa-miR-148a-3p | 1.896117 | 3.7221 | 5.14E-04 | 0.068612 | UP |
| 4 | hsa-miR-23a-3p | 1.003622 | 2.005028 | 8.26E-03 | 0.207538 | UP |
| 5 | hsa-miR-378g | 0.866517 | 1.823256 | 1.24E-03 | 0.110628 | UP |
| 6 | hsa-miR-30a-5p | 0.834559 | 1.783312 | 1.96E-02 | 0.308391 | UP |
| 7 | hsa-miR-93-5p | 0.693353 | 1.617037 | 1.68E-02 | 0.279933 | UP |
| 8 | hsa-miR-497-5p | 0.682571 | 1.604997 | 1.02E-02 | 0.207538 | UP |
| 9 | hsa-miR-200b-3p | 0.580053 | 1.494904 | 2.82E-02 | 0.350687 | UP |
| 10 | hsa-miR-16-5p | 0.555462 | 1.469639 | 5.44E-02 | 0.386215 | UP |
| 11 | hsa-miR-422a | -0.50323 | 1.41738 | 3.84E-02 | 0.381103 | DOWN |
| 12 | hsa-miR-644a | -0.47998 | 1.394723 | 2.08E-03 | 0.124605 | DOWN |
| 13 | hsa-miR-200a-3p | 0.445082 | 1.361391 | 5.88E-02 | 0.386215 | UP |
| 14 | hsa-miR-520a-5p | -0.40659 | 1.325552 | 3.27E-03 | 0.124605 | DOWN |
| 15 | hsa-miR-506-5p | -0.39559 | 1.315479 | 5.92E-03 | 0.197641 | DOWN |
| 16 | hsa-miR-1262 | 0.386293 | 1.307031 | 1.13E-02 | 0.207538 | UP |
| 17 | hsa-miR-4516 | 0.386214 | 1.306959 | 1.10E-02 | 0.207538 | UP |
| 18 | hsa-miR-1257 | -0.38522 | 1.306057 | 3.23E-03 | 0.124605 | DOWN |
| 19 | hsa-miR-1253 | -0.38439 | 1.305306 | 2.43E-02 | 0.350687 | DOWN |
| 20 | has-miR-503-5p | -0.36753 | 1.290142 | 8.58E-03 | 0.207538 | DOWN |
| 21 | has-miR-590-5p | -0.34374 | 1.269041 | 1.17E-02 | 0.207538 | DOWN |
| 22 | hsa-miR-513a-3p | 0.338847 | 1.264746 | 2.89E-02 | 0.350687 | UP |
| 23 | hsa-miR-143-3p | -0.32445 | 1.252189 | 3.59E-02 | 0.381103 | DOWN |
| 24 | hsa-miR-335-5p | 0.322646 | 1.250623 | 4.50E-02 | 0.386215 | UP |
| 25 | hsa-miR-185-5p | 0.317821 | 1.246446 | 3.05E-02 | 0.354176 | UP |
| 26 | hsa-miR-411-5p | -0.31197 | 1.241402 | 2.87E-02 | 0.350687 | DOWN |
| 27 | hsa-miR-371a-5p | -0.31176 | 1.241222 | 2.69E-02 | 0.350687 | DOWN |
| 28 | hsa-miR-592 | -0.31002 | 1.239725 | 4.28E-02 | 0.381103 | DOWN |
| 29 | hsa-miR-1305 | 0.307194 | 1.237299 | 4.23E-02 | 0.381103 | UP |
| 30 | hsa-miR-665 | -0.3071 | 1.237222 | 4.02E-02 | 0.381103 | DOWN |
| 31 | hsa-miR-767-5p | 0.307007 | 1.237139 | 3.44E-02 | 0.381103 | UP |
| 32 | hsa-miR-605-5p | -0.30419 | 1.234721 | 4.77E-02 | 0.386215 | DOWN |
| 33 | hsa-miR-526a | -0.30222 | 1.233041 | 8.86E-03 | 0.207538 | DOWN |
| 34 | hsa-miR-301a-5p | 0.295249 | 1.227096 | 4.77E-02 | 0.386215 | UP |
| 35 | hsa-miR-572 | -0.29462 | 1.226562 | 5.49E-02 | 0.386215 | DOWN |
| 36 | hsa-miR-891a-5p | 0.293378 | 1.225506 | 4.16E-02 | 0.381103 | UP |
| 37 | hsa-miR-603 | -0.27671 | 1.211433 | 5.07E-02 | 0.386215 | DOWN |
| 38 | hsa-miR-181b-2-3p | -0.25943 | 1.197003 | 5.24E-02 | 0.386215 | DOWN |
| 39 | hsa-miR-3065-5p | -0.25895 | 1.19661 | 5.77E-02 | 0.386215 | DOWN |
| 40 | hsa-miR-1307-5p | -0.25636 | 1.194459 | 5.86E-02 | 0.386215 | DOWN |
| 41 | hsa-miR-149-5p | -0.23239 | 1.174782 | 5.93E-02 | 0.386215 | DOWN |

**Suppl Table 2: Differentially expressed human milk exosomal miRNAs in mothers living with HIV-1 for 3 years**

| **Sr. No.** | **miRNA ID** | **Log Fold Change** | **Fold Change** | **p-value** | **Adj. p-value** | **Regulation** |
| --- | --- | --- | --- | --- | --- | --- |
| 1 | hsa-miR-320e | 2.99814 | 7.989734 | 2.01E-07 | 2.86E-05 | UP |
| 2 | hsa-miR-630 | 2.79309 | 6.931138 | 3.31E-17 | 9.42E-15 | UP |
| 3 | hsa-miR-30a-5p | 1.19366 | 2.287336 | 4.91E-06 | 0.00035 | UP |
| 4 | hsa-miR-378g | 0.96032 | 1.945747 | 1.49E-06 | 0.000142 | UP |
| 5 | hsa-miR-93-5p | 0.84462 | 1.795798 | 0.001015 | 0.048212 | UP |
| 6 | hsa-miR-23a-3p | 0.74021 | 1.670429 | 0.000385 | 0.02197 | UP |
| 7 | hsa-miR-200b-3p | 0.63601 | 1.55403 | 0.005171 | 0.210542 | UP |
| 8 | hsa-miR-126-3p | 0.58374 | 1.498736 | 0.00678 | 0.241539 | UP |
| 9 | hsa-miR-125b-5p | 0.54454 | 1.458565 | 0.025566 | 0.383462 | UP |
| 10 | hsa-miR-497-5p | 0.52191 | 1.435855 | 0.013069 | 0.353251 | UP |
| 11 | hsa-miR-16-5p | 0.50573 | 1.419848 | 0.022313 | 0.383462 | UP |
| 12 | hsa-miR-335-5p | 0.45799 | 1.373632 | 0.014574 | 0.353251 | UP |
| 13 | hsa-miR-301a-5p | 0.45773 | 1.373382 | 0.01865 | 0.379668 | UP |
| 14 | hsa-miR-26b-5p | 0.44300 | 1.359437 | 0.010608 | 0.335919 | UP |
| 15 | hsa-miR-200a-3p | 0.43390 | 1.350889 | 0.048097 | 0.502367 | UP |
| 16 | hsa-miR-423-5p | 0.42813 | 1.34549 | 0.044136 | 0.502367 | UP |
| 17 | hsa-miR-1305 | 0.42790 | 1.345278 | 0.020503 | 0.383462 | UP |
| 18 | hsa-miR-185-5p | 0.42646 | 1.343937 | 0.031616 | 0.409575 | UP |
| 19 | hsa-miR-526a | -0.36935 | 1.291776 | 0.049355 | 0.502367 | DOWN |
| 20 | hsa-miR-379-5p | -0.40747 | 1.326367 | 0.024573 | 0.383462 | DOWN |
| 21 | hsa-miR-574-5p | -0.40864 | 1.327438 | 0.039971 | 0.474658 | DOWN |
| 22 | hsa-miR-143-3p | -0.41213 | 1.330652 | 0.028015 | 0.383462 | DOWN |
| 23 | hsa-miR-603 | -0.41222 | 1.330734 | 0.039716 | 0.474658 | DOWN |
| 24 | hsa-miR-488-3p | -0.43444 | 1.351395 | 0.02764 | 0.383462 | DOWN |
| 25 | hsa-miR-3613-5p | -0.43540 | 1.352294 | 0.046911 | 0.502367 | DOWN |
| 26 | hsa-miR-506-5p | -0.46611 | 1.381381 | 0.028255 | 0.383462 | DOWN |
| 27 | hsa-miR-520a-5p | -0.51127 | 1.425313 | 0.014874 | 0.353251 | DOWN |
| 28 | hsa-miR-3065-3p | -0.55251 | 1.466642 | 0.016362 | 0.3587 | DOWN |

**Suppl Table 3: Differentially expressed human milk exosomal miRNAs in mothers living with HIV-1 for 4~15 years**

| **Sr. No.** | **miRNA ID** | **Log Fold Change** | **Fold Change** | **p-value** | **Adj. p-value** | **Regulation** |
| --- | --- | --- | --- | --- | --- | --- |
| 1 | hsa-miR-630 | 2.979356 | 7.886338 | 4.72E-05 | 0.012542 | UP |
| 2 | hsa-miR-320e | 2.691412 | 6.459455 | 0.008524 | 0.228975 | UP |
| 3 | hsa-miR-148a-3p | 1.949454 | 3.862282 | 0.000549 | 0.073056 | UP |
| 4 | hsa-miR-23a-3p | 1.088058 | 2.125877 | 0.007968 | 0.228975 | UP |
| 5 | hsa-miR-378g | 0.85723 | 1.811556 | 0.003007 | 0.159951 | UP |
| 6 | hsa-miR-497-5p | 0.722375 | 1.649896 | 0.00837 | 0.228975 | UP |
| 7 | hsa-miR-200b-3p | 0.55742 | 1.471636 | 0.043302 | 0.470288 | UP |
| 8 | hsa-miR-4516 | 0.418151 | 1.336214 | 0.008818 | 0.228975 | UP |
| 9 | hsa-miR-1262 | 0.402436 | 1.321737 | 0.010675 | 0.228975 | UP |
| 10 | hsa-miR-513a-3p | 0.372453 | 1.294552 | 0.022486 | 0.351833 | UP |
| 11 | hsa-miR-506-5p | -0.3675 | 1.290111 | 0.014512 | 0.27573 | Down |
| 12 | hsa-miR-503-5p | -0.37601 | 1.297746 | 0.009746 | 0.228975 | Down |
| 13 | hsa-miR-520a-5p | -0.37923 | 1.30065 | 0.007939 | 0.228975 | Down |
| 14 | hsa-miR-1253 | -0.40407 | 1.32324 | 0.025619 | 0.364158 | Down |
| 15 | hsa-miR-1257 | -0.43195 | 1.349059 | 0.00143 | 0.12677 | Down |
| 16 | hsa-miR-644a | -0.50783 | 1.421913 | 0.002051 | 0.136418 | Down |
| 17 | hsa-miR-422a | -0.54413 | 1.458139 | 0.037247 | 0.450354 | Down |

**Suppl Figure 1: Western Blot Confirmation of Human Milk Exosomes:** Western blotting for exosome-associated marker protein CD81. 10, 20, and 40 µg of proteins from either freshly isolated or human milk exosomes kept at room temperature (RT) for 2 days were loaded (Top and bottom Images with different exposure times).
