# Supplementary figures and images for: Expression profiling of human milk derived exosomal microRNAs and their targets in HIV-1 infected mothers
Source: Sci Rep. 2020 Jul 31;10:12931. doi: 10.1038/s41598-020-69799-x (PMC7395778; doi:10.1038/s41598-020-69799-x)

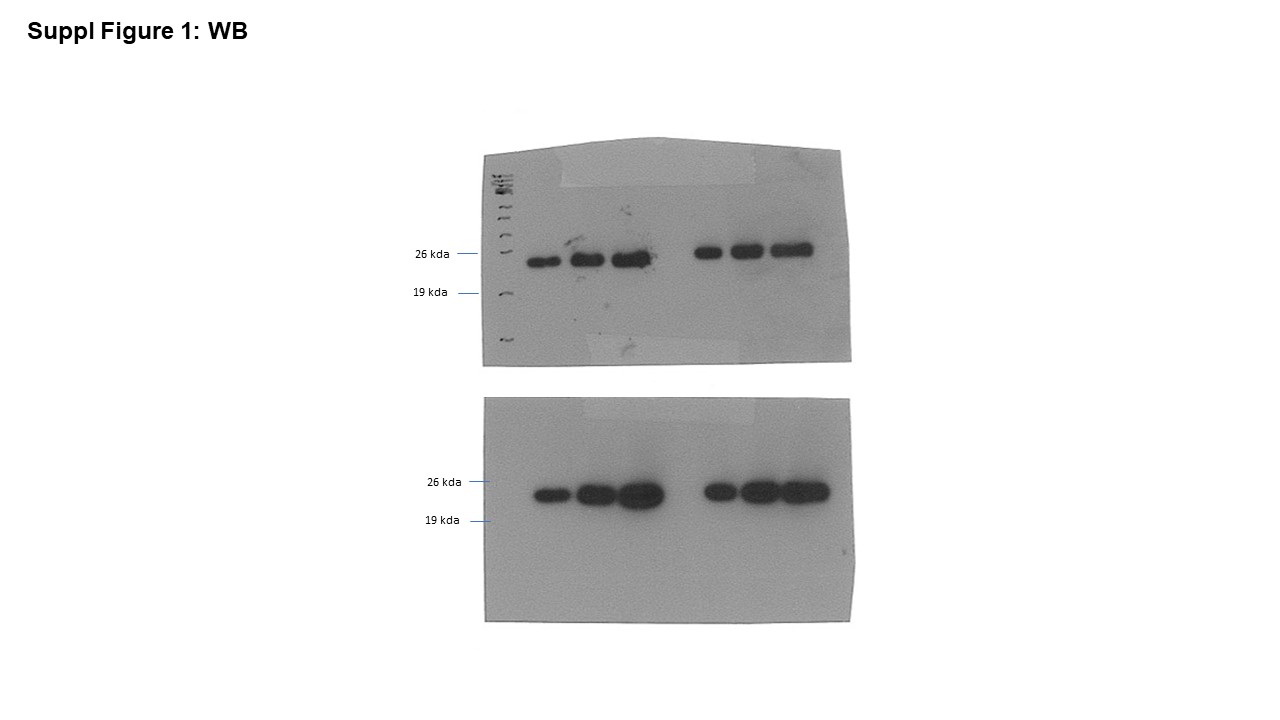

Supplement: Supplementary file 2 — Supplementary Figure S1 [file 41598_2020_69799_MOESM2_ESM.jpg]
